# National Association of Dental Faculties

**Published:** 1901-11

**Authors:** 


### NATIONAL ASSOCIATION OF DENTAL FACULTIES.

### REPORT OF THE FOREIGN RELATIONS COMMITTEE FOR THE YEAR 1900-1901.[1]

The past year has been an exceedingly active one for the Foreign Relations Committee, and the correspondence has been very large. We believe that the influence of the National Association of Dental Faculties has been materially extended during the year, and the good work that has been accomplished by it is becoming more widely known both at home and abroad.

The Association has given its Foreign Relations Committee

[1] Reported and adopted at the eighteenth annual meeting, held in Milwaukee, Wis., August, 1901.

jurisdiction in all foreign educational affairs that affect its interests. This we do not understand to mean that we can dictate what shall be the foreign policy to be followed, but that the committee may advise during the interim between the sessions, reporting its action for approval or disapproval at each annual meeting. This does not in any way interfere with the duties of the Ad Interim Committee, as the authority of the latter has never embraced matters in foreign countries. At the last annual meeting the committee presented a partial schedule of equivalents to be given for attendance on foreign courses of study. The Association accepted that and enacted that advanced standing in American schools should only be given foreign students in accordance therewith.

There is a rule of the Association that any legislation vitally affecting the members shall not go into effect for one year. This is a wise restriction, for the announcements are usually issued before the time of our annual meeting, and enactments that might be in conflict with the terms legally offered to students could not well be enforced. Your committee found that some foreign students had already been accepted by schools, and consideration given to foreign instruction which might be in conflict with the new regulations. It was therefore deemed best not to give any rulings affecting the annual term for 1900-1901.

But many loyal schools, those whose governors were most anxious to improve the standard of American professional education, have referred all their foreign applications to the committee. In this way it has been learned that foreign students have asked for advanced standing because of attendance, in some instances, on schools that had no existence whatever. Certificates have been presented from countries which have no dental legislation, and in which there is no semblance of a dental educational institution. They usually emanate from some private practitioner whose office is made to assume a sounding title. In other cases they pretend to be granted by some teaching hospital which has no official status.

Your committee has discovered that it is usual for the possessors of such doubtful credentials to write to a considerable number of schools to learn which, if any, will accept their certificates, and to find out whether some institution will not offer a special inducement. Each dean is assured that others will receive the applicant if he does not. The result is that all of those to whom application has been made are duly informed of the qualifications of suspected students and the probable terms on which they were accepted if the name is found on the lists of any school.

By this it is readily perceived how deans of colleges have been deceived in the past, and how the character of our American schools has been made to suffer for things over which they had no control. The Foreign Relations Committee is prepared to recommend a rating for any foreign school that will submit its curriculum of study and its preliminary requirements of education. This must be approved by the Association before becoming effective, and if our schools will govern themselves accordingly the old reproach that we give advanced standing on insufficient qualifications will be forever removed.

A very few schools have manifested some opposition to these regulations. Your committee has, even by some inconsiderate teachers, been accused of an attempt to dominate the colleges. We cannot conceive upon what grounds such a charge should be brought. The committee has done nothing save that which it was positively instructed to do. It has made no rules whatever. It has confined itself to recommending such legislation as it believed absolutely essential to the proper conduct of an educational institution, and if such recommendations have been given legislative enactment, it has tried to carry them into effect, but not otherwise. It has never exceeded its authority nor been unnecessarily aggressive in any of its proceedings. If there exists any reason for criticism of its action on other grounds than opposition to wholesome restraint and the desire to do that which of right ought not to be done, it will be very glad to have such instances pointed out, for its sole ambition has been to carry out the honest wishes of the parent body to which it is responsible.

### FRADULENT DENTAL DEGREES.

Last year, at the request of the committee, it was relieved of the task of endeavoring to suppress illegal and fraudulent degree-granting institutions, but, as it was already identified with the work, we found that we could not detach ourselves from it entirely. Letters and complaints were persistently directed to us instead of to the Law Committee, to which the subject had been referred. Besides, the diplomas which were sold by the fraudulent schools, and the principal attendance upon those which had a legal existence, but which are unrecognized and unaccepted here, was chiefly in and from foreign countries. Hence its consideration properly belonged to the Foreign Relations Committee, and we could not well refuse to receive the complaints and do what we could in the premises. In our last report we made public the fact that a number of the fraudulent institutions were suppressed and their conductors imprisoned. We hoped that this would practically close up all of them, but special circumstances have intervened to protect certain ones, and the work is not yet completed.

It is not generally known in this country that thousands of fraudulent diplomas have been sold abroad. Were it possible for foreigners to distinguish between the reputable and the disreputable schools, this would not so much matter, but the statutes of the State of Illinois, under which it is possible to incorporate degree-granting institutions which have practically no State supervision or responsibility whatever, and which with legal sanction are, under the great seal of the State, certified as lawfully organized colleges by the Secretary of State of Illinois, simply encouraged the fraud. By that certification of the Secretary of State the most unblushing impostures are placed apparently upon the same plane with reputable institutions, and foreigners are deprived of all means by which they can positively determine which is worthy recognition and which is not. As a consequence, some foreign governments have used this condition, either honestly or as a desirable pretext, to discriminate against all Americans, and have refused them permits to practise, and positively prohibited under heavy penalties the employment by any one of the American degree or title. This interdiction is spreading very fast, and, unless something is done to forestall it, soon the possession of an American diploma, whether legitimately or illegitimately obtained, will be a positive detriment to a practitioner. In fact, that is the case to-day in some parts of Germany. The influence of such enactments upon American educational affairs and upon the members of this Association may perhaps be imagined. Already prohibition is practically accomplished in Southern Germany, is impending in Northern Germany, has been commenced in France, in Italy, and in other countries, and there is sharply threatened a combination of all Europe against the American dental degree and the American dental school.

Much of this may, with a considerable degree of justice, be charged against the State of Illinois. Its own legislation has fostered the fraudulent schools that have brought this disgrace upon us. Its dental profession is not without responsibility. When has any body of its dentists put forth any special efforts to bring about a reform? The State has one of the best State dental societies in existence, with a large surplus in its treasury. For many years it has been a leader in thought, because within its membership has been found a great number of the very ablest men in dentistry; men who have done as much to advance dental practice as have any others. The papers read before that society have challenged the attention of the world. Many of the members must have known something of the opprobrious professional legislation upon the statute-books. Not a voice has been raised in denunciation of the condition. Not a word has been uttered, until at the last annual meeting a mild resolution deprecative of the infamous traffic was offered by one unconnected with either schools or boards.

The State Dental Examining Board of Illinois has practically recognized fraudulent and irregular colleges, schools either without any regular course of instruction or with but a very insufficient one, by admitting their students to the State examination and licensing them to practise, and by practically certifying to the regularity of institutions which every dentist in America knows, or should know, are conducted solely for whatever of revenue there may be in it. The law admits to the State examination for practice any one who asks for it, and the State Board of Dental Examiners has given the known fraudulent institutions a quasi status by admitting those holding their purchased diplomas to the examination, passing them, and giving them the certificate which makes of them regular and legal practitioners. This has been done under the law, but it is Illinois law, and the profession of the State is doing nothing to bring about a reform that professional decency imperatively demands. It is time that the many high-toned professional men of the State were aroused to the stern accountability to which they are liable to be called.

In directing attention to this, your committee must, in justice to the profession of the State, urge that in the opinion of some it has not been wise to admit that which has a real existence, in the hope that the State Board of Health and the medical profession might without scandal succeed in bettering the condition. Surely it must be now apparent to every one that the great work demands the most earnest efforts of every honest dentist of the State. The excellent schools of Chicago have not hesitated to step into the breach when educational interests and professional progress were threatened by the action of other State examining boards. Why should they not attempt a reform in the State law under which their own board acts?[1]

Last year your committee was able to report that the worst of the fraudulent schools of Chicago had been closed and that their conductors were in prison. That which was done was to a large degree the work of the State Board of Health of Illinois, which brought suit under the United States laws that forbid the use of United States mails for fraudulent purposes. In no other way could the general government at Washington interfere, because in all educational matters each State is autonomous, that being one of the reserved interests not delegated to the general government. The Board of Health being a State institution, it could commence proceedings in the name of the State, and use State funds for the prosecution of the criminals. It has been appealed to by your committee to take up the fraudulent issue of dental degrees, but the following letter will show that it purposes to confine its labors to the suppression of the sale of medical diplomas:

"STATE BOARD OF HEALTH, STATE OF ILLINOIS, SPRINGFIELD.
"OFFICE OF THE SECRETARY, July 13, 1901.

"DEAR SIR,—Your letter of the 3d was received during my absence in the North. In regard to the sale of dental diplomas in Illinois. I cannot give you the letter you desire, for this board is taking no steps whatever to break up the traffic in these degrees. Through the efforts of this board

---

[1] Subsequent to the reading of this report at a meeting of the National Association of Dental Faculties, about three thousand dollars was raised within an hour for prosecution of the work of reform. The National Dental Association afterwards appropriated one thousand dollars more. Before the close of the Milwaukee meetings, however, the Illinois dentists in attendance actively commenced the work on their own account, and within a week secured the appointment of a new State Dental Examining Board, while a part of the old board were placed under arrest for malfeasance in office and for general fraudulent practices. Proceedings were also very promptly commenced to annul the charters of certain irregular or fraudulent schools, and the prospect is very encouraging for the entire removal of the reproach that has so long rested upon them, thus verifying the confident predictions of the committee, that when the profession of the State were fully awakened to the real condition it would without delay be purified as by fire.—B.

the sale and barter of medical degrees has been entirely suppressed, and the persons who formerly made a business of selling degrees in medicine are now in jail.

"With the assistance of the governor of the State and a few medical men we succeeded in getting legislation passed in 1899 by means of which it is a very easy matter to summarily close up any institutions selling degrees in medicine, dentistry, or pharmacy. Under this law the notorious 'Metropolitan Medical College' has been closed.

"There seems to be no reason why the State Board of Dentistry cannot take action in the matter of sale of dental degrees. If the board chooses it can suppress within two weeks the institutions the 'diplomas' of which are sold in Munich or elsewhere. Why this board has taken no action on these lines I am unable to say. The State Board of Health sees no reason why it should assume duties which devolve upon another board. If any medical degrees are sold in this State, I am not aware of the fact. If proof of such sale be presented to this board, the institution or institutions in question can be closed within a month.

"Very truly yours,

"J. A. Egan,

"*Secretary.*"

It may thus be seen that we are thrown upon our own resources in the work of closing the institutions engaged in granting fraudulent or irregular dental degrees, and can look to the medical profession for no assistance. Your committee feels confident it can within a short time close up the last of the fraudulent schools if a sufficient sum of money can be placed at our disposal, and we are so advised by very competent legal counsel. We are prepared to submit a plan of procedure to this Association.

AMERICAN EDUCATIONAL AFFAIRS IN EUROPE.

During the past year professional events in Europe having relation to American educational affairs have crowded upon each other's heels in rapid succession. Partly as the result of the appointment of the Foreign Advisory Committees by the Association, and more especially through the action of United States governmental agents abroad, an attempt has been made to stem the tide which is so unjustly setting against us in Europe. The papers relating to such action were promptly sent to your committee. We recognized the fact that the purification of the stream must commence at the fountain-head. Practically no fraudulent degrees are sold in America; the countries of Europe are the sea into which the foul tide empties, and the sweetening of the waters cannot be effected there. It is in this country that the remedy must be applied, and until a healthy public and professional sentiment can be evoked here nothing can be done. The condition has existed for years, and it is constantly growing worse. A pest-hole cannot be cleansed until it is uncovered. A festering wound must be laid open that access can be obtained to its foulest depths. The community must be convinced from whence an infection proceeds before it will abolish the source. Few dentists are aware of what exists in this country. Any man knows that when the honest intelligence of our profession is fully awakened to any enormity, it will move heaven and earth if necessary to put an end to it.

Your committee seized upon the opportunity of the presentation of the most damning proofs coming from official sources to show to the dentists of America what really existed in their midst. Nine out of ten of them had little conception of the condition. When your committee, in its annual report for the year 1898, presented at Omaha, in part laid bare the grossness of the traffic in dental diplomas, the statement was received with incredulity. When that report had been softened in some of its expressions because a part of the committee feared it was exaggerated, it was even then a matter of amazement, and in no place more so than in the State of Illinois. But when inquiry revealed the fact that the half had scarcely been told, the deepest indignation was expressed. All the best of the general educational institutions of the State combined to bring about reform. In their wrath and righteous exasperation they went before the Legislature, and met with defeat, because their statements were disputed and their motives impugned by the men whom they attacked. They had no fully awakened public sentiment back of them. Very few were aware of the enormity of the fraud. Their facts were met by counterbalancing statements on the part of men whose honesty had not before been impeached; a flank movement was successfully manœuvred; they themselves were accused of improper motives, and the Legislature refused to act. Then an attack was made through the United States courts, which were not under the influence of public opinion, and they succeeded in breaking up a part of the iniquity and in getting through an amendment to the law under which it is possible to annul the charter of an openly fraudulent college. But new charters were easily obtained by the same men, and the work was recommenced under another name. The snake at best was scotched, and not killed. The time for another awakening seemed ripe, and your committee applied to the Secretary of State of the United States at Washington for permission to publish the official reports made to it by Consul Worman, of Munich, Germany. We believed that such publication, under the high sanction of the United States government, of official documents would challenge the attention of American people and greatly tend to produce a public sentiment powerful enough to sweep the fraudulent colleges from the face of the earth. Will it be believed?—from high places came public criticisms and protests against any open attempt to break up the infamous traffic which had seriously involved the reputation of every American school!

The name of Consul Worman has been mentioned. Your committee believes that his efforts to rehabilitate the American degree in Europe have been, and promise to be, of the greatest benefit to dentistry, and his work should be sustained by every one. Your committee has not been able to give him all the assistance it desired, because it was this year without the credit upon the treasurer of the Association that has been accorded it in the past, but it hopes that the good work may not be hindered by this obstacle in the future. Our national, our professional, our individual reputations are at stake. The good name of every member of this Association is in the balance, and our vindication from a foul blot upon our professional escutcheon must not be a matter of indifference. To assume that this is in the interests of antagonistic foreign governments, that it is doing their dirty police work, is to attempt to cover up and apologize for and justify the villany that is being done in our names; to assure complicity with the men who are trading on our good deeds, and who under cover of the high reputation of American dentistry, won by us, are endeavoring to foist upon foreign communities a counterfeit that must of necessity throw doubt upon the original.

### FOREIGN DENTAL SCHOOLS.

In the face of the fact that a most determined effort is being made in some foreign countries to break down the reputation of American dental schools, and to discredit all American professional education, and in the knowledge that not only are our courses refused any consideration, but sometimes made a pretext upon which to forbid Americans to enter upon practice, this Association cannot be accused of illiberality or of professional narrowness should it decline to accept foreign qualifications as a sufficient warrant for practice in this country. There should be some kind of reciprocity in professional affairs, and Americans ought not to be expected to extend all the professional courtesies granted. And yet, exact justice might, in the minds of many, demand that, irrespective of what may be done to us, we should be forgiving, and in return for the buffetings that we receive humbly expose the other cheek to the smiting hand. That course is perhaps highly Christian, but it is not quite in accordance with the impulses of an ordinary human nature. The man or the school that does not have sufficient of self-respect to maintain inalienable rights can scarcely expect to receive the consideration which may be honestly due.

But were this the only reason to be urged against the unquestioned acceptance of all foreign qualifications, we might justly be called churlish and professionally illiberal were we to exclude any one who asked our recognition. America was the first to establish any system of dental education. It embraced a full course of instruction, the whole of which must be covered within the walls of a duly chartered institution devoted to dental instruction. It was provided that all work leading to our special degree must be done under the direct supervision of qualified and accepted teachers. Recognizing the prosthetic department as one of the most important in dental practice, we insisted that it must have a scientific basis, and not be a matter of mere empiricism. We established the principle that our students must be under the pupilage of one who was acquainted with mechanical laws, and that the teaching of physical science should not be intrusted to possible charlatans. The instructor in mechanics must be responsible to the authority which granted the diploma or certificate of qualification.

The opposite course was pursued in founding the dental system of education in some other countries. Recognizing that many skilful mechanics were outside the pale of the fully qualified men, they practically excluded prosthetics from the college curriculum, classed mere mechanical skill as handicrafture, and permitted its instruction to be received at the hands of irresponsible men. They established a system of apprenticeship which in a manner bound out the student to a dental mechanic, who should give him instruction in one of the most important departments of dentistry. It could not be expected that we should accept such instruction as the equivalent for our full college courses. This condition was the most embarrassing question that came before your committee in the attempt to establish a system of equivalents. Our schools refuse to give to an American student any advanced standing for time spent in the laboratory or office of a practitioner who has not teaching experience and responsibility. The matriculant may have passed years in a dental office, but he must join the Freshman Class on entering our colleges. Our diplomas or certificates are only granted upon the completion of a definite scholastic course. Occasionally some one has urged that merit and knowledge and skill should be recognized wherever found, and without reference to their source. But that is the very pretext urged by the fraudulent and short-term schools for the granting of their honors after an incomplete course, they themselves conducting the examination, and being the sole judges of that skill and merit.

Why American colleges or college men should desire to shorten the usual term is past comprehension, for it is prejudicial both to their educational and their financial interests. A degree is granted as a reward for the completion of a full course. It is not a recognition of merit. No two men reap the same advantages from a given amount of instruction. One man graduates a skilled, dexterous practitioner, while another is much his inferior. But both have earned their diploma by having successfully completed a prescribed course of study. Many men in the profession do not comprehend this, and blame the schools because a graduate is not as clever and expert in his technical manipulation as the experienced practitioner. Our schools demand the successful completion of a definite course in mechanics. We cannot recognize the qualifications of any man who has not complied with a reasonable requirement that is demanded of our own graduates. We cannot accept the course of any school that does not require this, and your Foreign Relations Committee has not recommended as the equivalent for ours the certificates of any such schools. The most that we can do for those that accept the apprenticeship system as a part of their course is to give one year's advanced standing for the completion of a full and complete three or four years' pupilage with final graduation.

Under our present legislation it is illegal and irregular for any member of this Association to admit to its Senior Class any student who has not at least the following qualification:

Successful completion of two full terms in a dental school

*whose course has been accepted by this Association as a full equivalent for its own,* and who shall by that school be recommended for such advanced standing.

Admission to the second or Junior Class of any of our schools can only be permitted to those who have one of the following qualifications:

(1) Successful completion of one full term in a dental school whose course has been accepted by this Association as a full equivalent for its own course, the student being by that school recommended for such advanced standing.

(2) Successful completion of the full course of some regular and duly accepted medical school, and graduation with the degree of Doctor of Medicine.

No partial courses are accepted, nor those spent in a school not fully and definitely recognized by this Association. Surely we cannot grant more than this to those making application from foreign countries while denying it to our own people.

This principle has governed the Foreign Relations Committee in making its recommendations for the recognition of foreign schools. There have been urgent requests for such recognition, but your committee has not felt itself at liberty to recommend what is not granted to our own schools and people. If any foreign school will demonstrate that its curriculum of study is the full equivalent of our own, and that it has complied with the statute of minimum requirements established by this Association at its last annual meeting, your committee will be prepared to examine its claims and to recommend such action to this Association as the course of study seems to warrant.

Your committee, in conclusion, points with no ordinary pride to what has been accomplished within the past five years as the result of an attempt to regulate our relations with foreign schools and foreign students, and to the high professional ground on which we now stand. There should be no further complaints, on the one hand that we accept unqualified men from abroad, or on the other that foreigners can come here and, without going through the full course demanded of American students, carry off our honors and claim to be American dentists, the colleagues of those who have completed our full curriculum of a broad course of dental study.

The foreign advisory boards, appointed with the approval of this Association, have proved to be useful auxiliaries in the carrying out of our system of education. In Europe they have completed an organization, and will henceforth work together in harmony. They must exercise an important and wide influence in educational affairs, and their action cannot but be good. They will guard the interests of those holding the American degree, and help to prevent it from being unworthily conferred. Your committee has made some further appointments in countries heretofore unrepresented, which it reports for approval. It is very much to be desired that at each of our annual meetings representatives from these foreign advisory boards should be in attendance whenever possible, and we recommend the enactment of a standing resolution giving to such regular representatives a seat in our meetings with the usual privileges of the floor.

REPORT CONCERNING FOREIGN EQUIVALENTS AS AMENDED FOR THE YEAR 1901.

Were your committee to follow the precedent set by most foreign countries no consideration would be given to their qualifications. Although America set an example to all the world in establishing a definite curriculum of instruction for dentists, in organizing schools for their theoretical and practical training, thereby erecting into a recognized profession or specialty that which previously was mainly empiricism and charlatanry, no official recognition of its special curriculum has ever been given by the dentists of foreign countries, although in great numbers they have attended our schools to obtain the advantages offered by that curriculum.

Your committee believes it to be neither fraternal, professional, nor just to adopt the same course, but thinks it both expedient and right to extend proper recognition to whatever can be received as an equivalent for our own courses. It must not be forgotten, however, that the system of dental instruction in Europe varies very widely from that of our special American schools. Instruction separate from that afforded by the medical schools or universities is very rare, and the practical training which forms a part of our curriculum is usually given by private preceptors. Your committee does not feel at liberty to recommend the acceptance of an oral and theoretical course as the equivalent for one including practical work. We cannot believe that the certificates of private and irresponsible practitioners can by us be accepted at any part of a college course, and hence we have given them little consideration.

*Australia.*

A very complete report from the various colonies of Australia and New Zealand has been made by the advisory board appointed for those countries. It would appear that in most of the colonies there is no dental legislation, but Victoria has lately secured a law analogous to that of England, and in Melbourne a dental school has been organized whose curriculum, from the partial syllabus furnished, seems to be a comparatively broad one. The institution has been but recently established, and your committee has been unable as yet positively to determine whether in all respects it complies with our minimum requirements. When this shall have been definitely determined, we shall be prepared to recommend to this body some proper action.

In the provinces of Western Australia and Tasmania no dental legislation has been secured.

There is a dental law in New Zealand, and the member of the advisory board from that province has furnished your committee with an abstract of it. There are no dental schools in the province.

*Switzerland.*

This is a republic analogous to our own country in some respects, the federal union being composed of separate cantons. There are some excellent universities which offer certain facilities for dental study, but their practical instruction, we believe, cannot be accepted as an equivalent for that offered by American dental colleges. Your committee recommends that holders of the Swiss national diploma be given one year's advanced standing in the schools of this Association, but that no consideration be at present extended to holders of the cantonal qualifications.

*Spain.*

The Spanish requirements in medicine are very high, but your committee has not learned that there are any dental schools, or dental departments of universities, whose course of instruction can be accepted as the full equivalent for the instruction given in American dental colleges.

*France.*

In accordance with the recommendations of the advisory board for this country, your committee recommends as follows:

That one year's advanced standing be given to students possessing the French government diploma of "Chirurgien Dentiste" who have completed the three years' course in either the "Ecole Dentaire de Paris" or the "Ecole Odontotechnique," and that the same consideration be given the French diploma of Doctor of Medicine.

That in all cases the American preliminary examinations as to educational requirements be demanded, and that a sufficient acquaintance with the English language to enable the student to comprehend lectures be an essential.

### *Germany and Austria.*

Your committee recommends that students speaking the English language, who have taken the full dental course in German or Austrian universities, be eligible for reception in the second-year classes of American dental colleges, provided it be shown that they have had at least two semesters of competent college instruction in practical laboratory and operative work.

### *Italy.*

There are, we believe, no schools in Italy which have courses that can be accepted as equivalent to those of our American dental schools. The instruction given in the medical schools your committee believes to be too exclusively general in its character to form an acceptable course in dentistry for American students.

### *Holland and Belgium.*

In these countries the title of dentist is obtained by passing a practical examination in the theory and practice of dentistry. There are no separate dental schools, and we are not sufficiently informed of the comprehensiveness of the syllabi of the universities to offer any recommendations concerning them.

### *Great Britain.*

Your committee recommends that all students who shall have finished the complete course in any recognized English, Irish, or Scotch dental school or hospital, shall be eligible for reception as second-year students in American dental colleges upon proof of their having taken as a part of such foreign course two years of instruction in a properly equipped dental laboratory and dental infirmary connected or affiliated with such dental school or hospital, and which requires the successful completion of the work deemed essential by recognized American schools, as formulated in the minimum requirements for foreign dental schools accompanying this report. We further recommend that for the present no consideration be given to partial courses in any of the dental schools of Great Britain.

### *Denmark, Sweden, and Norway.*

Sweden has one dental school, which is the Dental Department of the Caroline Medico-Chirurgical Institute of Stockholm. Instruction is given by five professors of the Medical Department, and there are three dental professors, occupying respectively the chairs of Dental Surgery, Operative Dentistry, and Dental Prosthetics and Orthodontia. From the assurances given, your committee believes that its graduates should be permitted to enter the second-year class of recognized American dental colleges, provided they shall have complied with our requirements concerning mechanical laboratory work.

Your committee has not sufficient knowledge concerning any school in Denmark or Norway to warrant further recommendations at present.

### *Japan.*

There is one dental school in Japan. It confers no degree, but gives a certificate which entitles the holder to government examination, the same as if he had studied with some practising dentist. As the instruction is personal and the school is quite irresponsible, your committee believes that no consideration can be given to those completing its courses.

### *Mexico.*

There is a medical school in the City of Mexico which purports to give dental instruction. Your committee cannot learn that it is of such a character as will enable it to be accepted as the equivalent for a course in an American dental college.

### *Canada.*

There is but one school in the Dominion, so far as your committee is aware, whose courses can be accepted as an equivalent for those of our own colleges, and that is at present a member of this body, so that it requires no special ruling.

### *Other Foreign Countries.*

Concerning the educational status of other nations, your committee is not in possession of sufficiently definite information to warrant any action whatever. We have no knowledge of the existence of any courses of instruction which can be accepted as an equivalent for the courses in the institutions having membership in this body, and therefore advanced standing in our schools cannot in justice to our own students be granted save in the instances above enumerated.

### REPORT CONCERNING MINIMUM REQUIREMENTS.

That a proper standard may be adopted by which the relative value of the courses in foreign dental schools whose students offer them as equivalents for a part of the instruction given in the colleges of the Association may be determined, your committee recommends the approval of the following as the minimum of requirements demanded:

1. The college must require of matriculants a preliminary education which is the full equivalent of that demanded by the schools of this Association.

2. The college must demand of students full attendance upon at least three full annual courses (not semesters) of lectures of not less than seven calendar months each in separate years, covering all the studies proper to a full dental curriculum.

3. The college must possess a bacteriological laboratory, with sufficient of equipment for instruction in a competent course in bacteriology, which must form a part of its curriculum of study.

4. The same must be required in chemistry, histology, and pathology.

5. There must be a technic laboratory in which shall be taught the proper manipulation for the insertion of all kinds of fillings for teeth, the preparation and filling of the roots of teeth, the tempering and shaping of instruments, the drawing of wire and tubing for cases in orthodontia, and the cutting of bolts and nuts.

6. There must be prosthetic laboratories sufficiently equipped for teaching all kinds of prosthetic work, and the construction of all the approved prosthetic appliances.

7. There must be a sufficiently equipped laboratory for instruction in making crowns and bridges, and the construction of appliances used in orthodontia.

8. There must be a properly equipped infirmary or surgery for the reception of patients, upon whom each and every student shall be required individually to perform all and enough of the operations necessary in dental practice thoroughly to qualify him for the successful pursuance of his profession.

9. Complete records of the work done by each student, of his attainments at sufficient and full examination in each subject of the curriculum of study, of his attendance and deportment during the course, must be permanently kept.

10. No credit must be allowed for any work not done under the immediate supervision of instructors connected with or especially approved by the college, and who are in direct affiliation with the faculty.

FOREIGN ADVISORY BOARDS.

The following is a list of the countries for which advisory boards have been designated, and the appointments and nominations so far as made:

| Country. | Name. | College. | Post-Office Address. |
|---|---|---|---|
| Great Britain. | Wm. Mitchell, D.D.S. | Univ. of Michigan. | 39 Upper Brook Street, London, England. |
| Great Britain. | W. E. Royce, D.D.S | Phila. Dental Coll. | 2 Lonsdale Gardens, Tunbridge Wells, England. |
| Great Britain. | B. J. Bonnell. | ...................... | 94 Cornwall Gardens, S. Kensington, London. |
| Holland and Belgium. | J. E. Grevers, D.D.S. | ...................... | 13 Oude Turfmarkt, Amsterdam, Holland, |
| Holland and Belgium. | Ed. Rosenthal, D.D.S. | Harvard University. | 19 Boul. du Regent, Brussels, Belgium. |
| Holland and Belgium. | C. Vander Hoeven, D.D.S. | ...................... | Der Haag. |
| Denmark, Sweden, and Norway. | Elof Förberg, D.D.S. | Phila. Dental Coll. | Sturegatan 24, Stockholm, Sweden. |
| Denmark, Sweden, and Norway. | S. S. Andersen, D.D.S. | Univ. Pennsylvania. | Christiania, Norway. |
| Denmark, Sweden, and Norway. | L. P. Vorslund-Kjaer, D.D.S. | Phila. Dental Coll. | Copenhagen, Denmark. |
| Russia. | H. V. Wollison, D.D.S. | N. Y. Coll. Dentistry. | 10 Quai de l'Amaranti, S. Petersburg, Russia. |
| Russia. | Theo. Weber, D.D.S. | N. Y. Coll. Dentistry. | Helsingfors, Finland. |
| Russia. | Geo. Th. Berger, D.D.S. | Phila. Dental Coll. | St. Petersburg, Russia. |
| Germany. | W. D. Miller, D.D.S. | Univ. Pennsylvania. | Victoriastrasse 30, Berlin, Germany. |
| Germany. | C. F. W. Bödecker, D.D.S. | N. Y. Coll. Dentistry. | 55 Unter den Linden, Berlin, Germany. |
| Germany. | Friedrich Hesse, D.D.S. | N. Y. Coll. Dentistry. | Goethe Str. 6, Leipsig, Germany. |
| Austria and Hungary. | Otto Szigmondi, M.D., Ph.D. | University Vienna. | Schmerlingplatz 2, Vienna I, Austria. |
| Austria and Hungary. | Rudolf Weiser, M.D., Ph. D. | University Vienna. | Frankgasse 2, Vienna, IX, Austria. |
| Austria and Hungary. | Dr. Jos. Arkövy. | Univ. Budapest. | Vaczi-utca, Budapest, Hungary. |

| COUNTRY. | NAME. | COLLEGE. | POST-OFFICE ADDRESS. |
|---|---|---|---|
| Italy and Greece. | Albert T. Webb, D.D.S. | Univ. Pennsylvania. | 87 Via Nazionale, Rome, Italy. |
| Italy and Greece. | Tullio, Avanzi. | Nominated. | ........................ |
| Italy and Greece. | A. V. Elliott, D.D.S. | Univ. of Michigan. | 10 Via Tornabuoni, Florence, Italy. |
| France. | J. H. Spaulding, D.D.S. | Univ. of Minnesota. | 39 Boul. Malesherbes, Paris, France. |
| France. | George B. Hayes, D.D.S. | Univ. of Michigan. | Paris, France. |
| France. | G. A. Roussell, D.D.S. | N. Y. Coll. Dentistry. | 74 Boul. Haussman, Paris, France. |
| Spain and Portugal. | R. H. Portuondo, D.D.S. | Univ. Pennsylvania. | Paseo de Recoletos 3, Madrid, Spain. |
| Spain and Portugal. | Florestan Aguilar, D.D.S. | Phila. Dental Coll. | Serrano 5, Madrid, Spain. |
| Spain and Portugal. | T. J. Thomas, D.D.S. | ........................ | Bilboa, Spain. |
| Switzerland and Turkey. | L. C. Bryan, D.D.S. | Boston Dental Coll. | St. Alban Anlage, Basel, Switzerland. |
| Switzerland and Turkey. | Theo. Frick, D.D.S. | Univ. Pennsylvania. | 14 Tonhallenstrasse, Zurich, Switzerland. |
| Switzerland and Turkey. | Paul J. Guye, D.D.S. | Penn. Dental Coll. | 12 Rue de Candolle, Geneva, Switzerland. |
| Japan, China, and India. | ........................ | ........................ | ........................ |
| Japan, China, and India. | J. Ward Hall, D.D.S. | ........................ | Shanghai, China. |
| Japan, China, and India. | ........................ | ........................ | ........................ |
| Australia & N. Zealand. | Alfred Burne, D.D.S. | Phila. Dental Coll. | 1 Lyon Terrace, Liverpool Street, Sydney. |
| Australia & N. Zealand. | Dr. A. P. Merrill. | Phila. Dental Coll. | 52 Collins Street, Melbourne. |
| Australia & N. Zealand. | Herbert Cox, D.D.S. | Univ. of Michigan. | 216 Queen Street, Auckland, New Zealand. |
| Cuba & W. India Islands. | ........................ | ........................ | ........................ |
| Cuba & W. India Islands. | Rice R. Buchanan, D.D.S. | ........................ | 47 San Francisco Street, San Juan, Porto Rico. |
| Cuba & W. India Islands. | A. E. Mascort. | Nominated. | Havana, Cuba. |
| Mexico & Cent. America. | H. W. F. Buttner. | Nominated. | City of Mexico. |
| Mexico & Cent. America. | J. W. Purnell. | Nominated. | Merida, Yucatan. |
| Mexico & Cent. America. | J. Hunter. | Nominated. | Puerto Cortez, Honduras. |
| Venez., Colom., & Ecua'r. | Manuel V. Toledo. | Nominated. | Caracas, Venezuela. |
| Venez., Colom., & Ecua'r. | J. R. Martinez. | Nominated. | Guayaquil, Ecuador. |
| Venez., Colom., & Ecua'r. | ........................ | ........................ | ........................ |
| Peru, Bolivia, and Chili. | Charles B. Davies, D.D.S. | Penn. Dental Coll. | 49 Plaza Anibal Pinto, Valparaiso, Chili. |
| Peru, Bolivia, and Chili. | S. R. Salazar, D.D.S. | Chicago Coll. Dental Surgery. | Lima, Peru. |
| Peru, Bolivia, and Chili. | C. W. Sparrock, D.D.S. | Nominated. | Lima, Peru. |
| Brazil and Guiana. | J. L. Fordham. | Nominated. | Rio de Janeiro, Brazil. |
| Brazil and Guiana. | Julius Weinburger. | Nominated. | Para, Brazil. |
| Brazil and Guiana. | ........................ | ........................ | ........................ |
| Argentine, Para., & Uru. | J. S. Burnett. | Nominated. | Salto, Uruguay. |
| Argentine, Para., & Uru. | J. C. Macartney. | Nominated. | Montevideo, Uruguay. |
| Argentine, Para., & Uru. | ........................ | ........................ | ........................ |

MEMBERSHIP OF THE NATIONAL ASSOCIATION OF DENTAL FACULTIES AT ADJOURNMENT, JULY, 1901.

The following is a list of the dental colleges of America which at the present time are members of the National Association of

Dental Faculties, whose diplomas and tickets alone are recognized and received by the members of the Association:

*Alabama.*—Birmingham: Birmingham Dental College.

*California.*—San Francisco: Dental Department, College of Physicians and Surgeons; University of California, College of Dentistry. Los Angeles: College of Dentistry, University of Southern California.

*Colorado.*—Denver: Colorado College of Dental Surgery.

*District of Columbia.*—Washington: Dental Department, National University; Dental Department, Columbian University; Dental Department, Howard University; Georgetown University, Dental Department.

*Georgia.*—Atlanta: Atlanta Dental College; Southern Dental College.

*Illinois.*—Chicago: Chicago College of Dental Surgery; College of Dentistry, University of Illinois; Northwestern University Dental School.

*Indiana.*—Indianapolis: Central College of Dentistry; Indiana Dental College.

*Iowa.*—Iowa City: University of Iowa, College of Dentistry. Keokuk: Keokuk Dental College, Dental Department of Keokuk Medical College.

*Kentucky.*—Louisville: Louisville College of Dentistry, Department of Central University of Kentucky.

*Louisiana.*—New Orleans: New Orleans College of Dentistry.

*Maryland.*—Baltimore: Baltimore College of Dental Surgery; Baltimore Medical College, Dental Department; Dental Department, University of Maryland.

*Massachusetts.*—Boston: Dental School of Harvard University; Tuft's College Dental School.

*Michigan.*—Ann Arbor: Dental College, University of Michigan. Detroit: Dental Department, Detroit Medical College.

*Minnesota.*—Minneapolis: College of Dentistry, Department of Medicine, University of Minnesota.

*Missouri.*—Kansas City: Kansas City Dental College; Western Dental College. St. Louis: Marion-Sims Dental College; Missouri Dental College, Dental Department of Washington University.

*Nebraska.*—Omaha: Dental Department, University of Omaha.

*New York.*—New York: New York College of Dentistry; New

York Dental School. Buffalo: University of Buffalo, Dental Department.

*Ohio.*—Cincinnati: Cincinnati College of Dental Surgery; Ohio College of Dental Surgery. Columbus: Ohio Medical University, Dental Department. Cleveland: Western Reserve University, Dental Department.

*Oregon.*—Portland: North Pacific Dental College.

*Pennsylvania.*—Philadelphia: Dental Department, University of Pennsylvania; Medico-Chirurgical College of Philadelphia, Department of Dentistry; Pennsylvania College of Dental Surgery; Philadelphia Dental College. Pittsburg: Pittsburg Dental College, Department of Western University of Pennsylvania.

*Tennessee.*—Nashville: Dental Department, University of Tennessee; Department of Dentistry of Vanderbilt University; School of Dentistry of Meharry Medical College, Department of Central Tennessee College.

*Virginia.*—Richmond: University College of Medicine and Surgery, Dental Department.

*Wisconsin.*—Milwaukee: Milwaukee Medical College, Dental Department.

*Canada.*—Toronto: Royal College of Dental Surgeons of Ontario.

William C. Barrett,
208 Franklin Street, Buffalo, N. Y.,

John D. Patterson,
Ninth and Walnut Streets, Kansas City, Mo.,

Truman W. Brophy,
126 State Street, Chicago, Ill.,

M. W. Foster,
9 W. Fayette Street, Baltimore, Md.,

Eugene H. Smith,
283 Dartmouth Street, Boston, Mass.,

*Foreign Relations Committee.*

